# Identification and immunological characterization of cuproptosis-related molecular clusters in cardioembolic stroke

**DOI:** 10.1097/MD.0000000000043747

**Published:** 2025-08-15

**Authors:** Dandan Zhang, Qian Zhang, Yan Wang, Ning Zhang

**Affiliations:** a People’s Hospital of Chongqing Banan District, Chongqing, China; b Heilongjiang University of Chinese Medicine, Harbin, China.

**Keywords:** bioinformatics, cardioembolic stroke, cuproptosis, differentially expressed cuproptosis-associated genes, molecular clusters

## Abstract

This study explored the molecular patterns and diagnostic biomarkers associated with cuproptosis in cardioembolic stroke (CES) using bioinformatics tools. GSE58294 expression profile data were downloaded from the Gene Expression Synthesis Database as a training dataset, and cuproptosis-related genes were extracted for analysis. We identified differentially expressed cuproptosis-associated genes (DECAGs) between CES and control samples. In total, 11 DECAGs (*MTF1*, *NFE2L2*, *DLD*, *ATP7B*, *ATP7A*, *SLC31A1*, *PDHA1*, *CDKN2A*, *DLS*, *DLAT*, *PDHB*) and activated immune responses differed significantly between CES patients and non-CES controls. Two molecular clusters associated with cuproptosis were discerned in CES. Immunoinfiltration analysis revealed significant immunoheterogeneity among the different molecular clusters, and Cluster1 showed a relatively high level of immunoinfiltration. Weighted gene co-expression network analysis revealed 819 key genes related to CES, 320 key genes related to CES typing, and 37 core intersection genes were identified by Venn analysis to construct a training model. The generalized linear model showed satisfactory performance based on 2 external validation datasets. Within this model, 5 genes (*FLT3LG*, *MAL*, *TNFRSF25*, *CASP5*, and *MAN1C1*) were identified as the most significantly associated with CES characteristics. Clinical application columns were established based on the expression levels of 5 CES characteristic genes. Decision curve analysis and correction curves showed that the results had good prediction accuracy. The present findings highlight the crucial role of cuproptosis in the development and diagnosis of CES, indicating its potential as a key factor in understanding and identifying CES.

## 1. Introduction

Approximately 70% of cardioembolic stroke (CES) cases are caused by atrial fibrillation (AF). The incidence of AF increases gradually with age, rising from 0.1% in individuals aged 55 years to 10% in patients aged 80 years.^[1–3]^ Therefore, the proportion of AF patients over 80 years was higher among those with acute ischemic stroke,^[4]^ making AF a critical stroke cause in this older population.^[5–7]^ However, the pathogenesis of CES has not yet been elucidated. Existing studies have shown,^[8]^ that high concentrations of copper ions have a strong correlation with cardiovascular diseases. Copper is an auxiliary substance of human essential enzymes, which plays an important role in human life activities.^[9]^ The amount of copper in normal cells is very small, and its function is to prevent the accumulation of harmful copper ions in cells and maintain the stability of the amount of copper in cells to a certain extent.^[10,11]^ Previous studies have shown that copper death is related to mitochondrial respiration. In this process, copper directly combines with the lipid components in the ternary acid ring, thereby causing the aggregation of fatty acylated proteins, resulting in the loss of iron–sulfur tufting proteins and causing protein toxic stress resulting in cell death.^[12]^

Cuproptosis plays an important role in the occurrence and progression of many diseases, including cancer, cardiovascular diseases, and neurodegenerative diseases.^[13]^ In addition, cuproptosis may be a new therapeutic target for stroke.^[14]^ Copper ions inhibit vascular cell function by increasing prothrombin level, exacerbating ischemic stroke conditions.^[15]^ and high concentrations of copper ions may lead to cell apoptosis and tissue damage after ischemia reperfusion.^[16]^ After CES leads to cerebral ischemia, neuronal cell death increases, leading to local and systemic inflammation, consequently resulting in inflammatory ischemia–reperfusion injury.^[17,18]^ Therefore, copper death can induce nerve cell apoptosis and aggravate the development of cardiovascular disease and stroke.

Therefore, bioinformatics techniques were used to screen for differentially expressed cuproptosis-associated genes (DECAGs) between samples with CES and control samples. This study aimed to explore the associated molecular patterns and potential diagnostic biomarkers of DECAGs in CES. To predict the potential diagnostic value of DECAGs in CES, samples with CES were classified using the consensus clustering method, and weighted gene co-expression network analysis (WGCNA) was performed to identify CES core genes. In addition, by comparing multiple machine learning algorithms, predictive models for CES samples with different molecular clusters were established. Our findings may help enhance the early diagnosis and tailor individualized treatment approaches for samples with CES.

## 2. Materials and methods

### 2.1. Data acquisition and pre-processing

A microarray dataset (GSE58294) associated with CES was obtained from the Gene Expression Omnibus (www.ncbi.nlm.nih.gov/geo). The dataset was annotated using the Practical Extraction and Reporting Language (https://www.r-project.org/, version4.1.0) script. Using the GPL570 platform, the GSE58294 dataset, involving blood samples from 23 control samples and 69 CES samples with CES, was further analyzed. The original gene expression profiles of these Gene Expression Omnibus datasets were processed using the Limma package in R language to obtain the corrected cuproptosis gene expression levels.

### 2.2. Analysis of the correlation between cuproptosis gene variability and immune infiltration

To further demonstrate the DECAGs between the control and trial groups, the expression levels of cuproptosis-related genes were extracted from the CES dataset using the “limma” R language package. Genes with significant expression differences associated with CES cuproptosis were obtained; the “reshape” R language package was used to draw heat maps, and the “ggpubr” R language package was used to draw box plots. The immune infiltration of CES genes was analyzed using the “CIBERSORT” R language package, and the box plot of the genes was drawn using the “ggpubr” R language package. The “limma” R language package was used to map the chromosomal location loop of cuproptosis-related genes. Using the “corrplot” R language package, DECAGs correlation and loop graphs were drawn. To analyze the correlation between DECAGs in immune cells and function, the “limma” R language package was used to analyze 2 result files of immune cell infiltration and the DECAGs expression matrix in CES, and a correlation heat map was constructed.

### 2.3. Unsupervised clustering of CES samples

Consensus clustering is a method of selecting the optimal number of clusters based on various clustering algorithms.^[19]^ In this study, the “ConsensusClusterPlus” R language package was used to divide CES samples into different subgroups based on the amount of DECAGs expression. The “ConsensusClusterPlus” command was executed 1000 times to ensure classification stability. The number of clusters (k) was determined using a uniform clustering cumulative distribution function. In addition, t-distributed stochastic neighbor embedding is a dimensionality reduction method that can reveal population stratification at different scales.^[20]^ This method was used to verify the classification performance based on the above DECAGs expression profile.

### 2.4. Analysis of cuproptosis-related immunoinfiltration and gene set enrichment analysis enrichment between subgroups

The result files of typing and immune cell infiltration were integrated using the “Limma” R language package. The “ggpubr” R language package was employed to draw a box diagram. The “ssGSEA” R language package was used to correct the scores of genotyping genes and analyze the pathway differences. In addition, the “ggpubr” R language package was used to select the top 10 channels to draw a histogram.

### 2.5. WGCNA

WGCNA can be used to investigate correlations in biochip samples and identify suitable biomarkers or therapeutic targets in different biological environments.^[21]^ The “WGCNA” R language was used to screen CES genes and core modules of typing and expressed genes. The top 25% of genes with the greatest fluctuation were selected for WGCNA, and an adjacency matrix (topological overlap matrix [TOM]) was constructed to describe the correlation strength between nodes. The adjacency matrix was transformed into a TOM, and the similarity between nodes was described quantitatively. Hierarchical clustering was performed to identify modules with a minimum of 100 genes. Each module was randomly assigned a color. Module signature genes represent the global gene expression profiles in each module. Module importance indicates the correlation between the module and the disease gene, representing the closeness between the genes of the module. Genetic significance was defined as the correlation between a gene and its clinical phenotype.

### 2.6. Construction of predictive models based on multiple machine learning methods

Support vector machine (SVM) and random forest (RF) models were built using the “randomForest” and “kernlab” R packages. Generalized linear model (GLM) and the eXtreme gradient boosting (XGB) were built using the “xgboost” and “gplot2” R language. The “VennDiagram” R package was used to construct intersection genes of CES disease genes and subtypes, with intersection genes used as explanatory variables and CES genes used as response variables. To obtain the most suitable model for training, box plots of the receiver operating characteristic curve (ROC), inverse cumulative distribution, and residual were plotted. Each model ranked the genes in order of importance and selected the first 5 genes. Using the “rms” R language, a nomogram was constructed to calculate a score for each item by projecting a small range (point) based on the characteristics of each variable of the patient. The scores for each item were added to calculate the total value; the higher the total value, the higher the probability of developing a CES. Calibration curves and decision curve analysis were used to determine the accuracy of the models.

### 2.7. Ethics and dissemination

Ethical approval was not required for this bioinformatic study of cuproptosis as we did not use data related to any individual patients. The final version of the manuscript will be published in a peer-reviewed scientific journal or at conferences to provide evidence-based medical support on CES and its genetics and molecular pathogenesis as well as new therapeutic targets for clinical workers. The dataset will be made freely available.

## 3. Results

**Figure 1.**
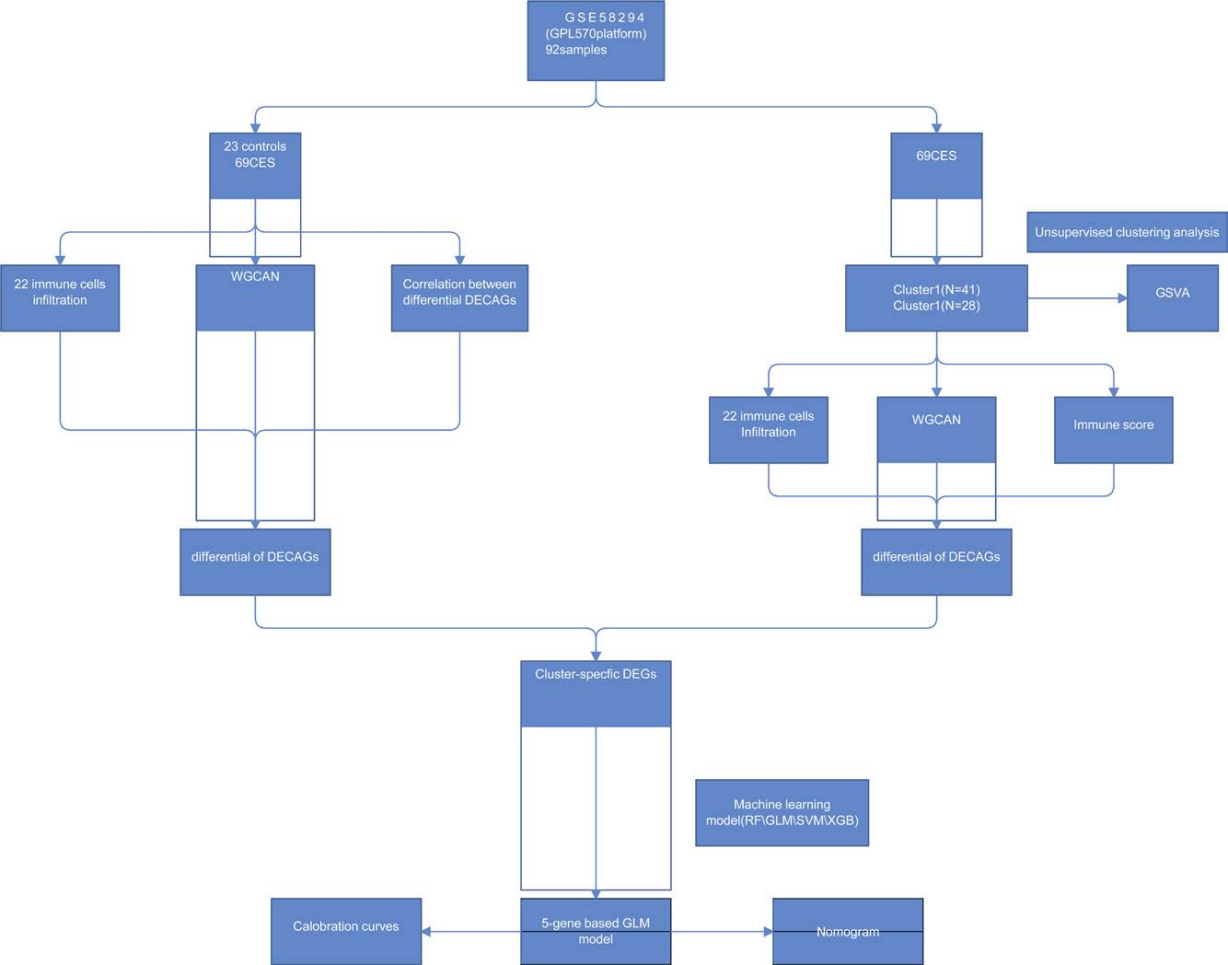
The study flow chart.

To clarify the biological functions of cuproptosis regulators in the occurrence and progression of CES, we first systematically evaluated the expression profiles of 11 DECAGs between CES samples and non-CES controls using the GSE58294 dataset. A detailed flowchart of the study process is shown in Figure 1.

### 3.1. Analysis of cuproptosis gene variability associated with CES

**Figure 2.**
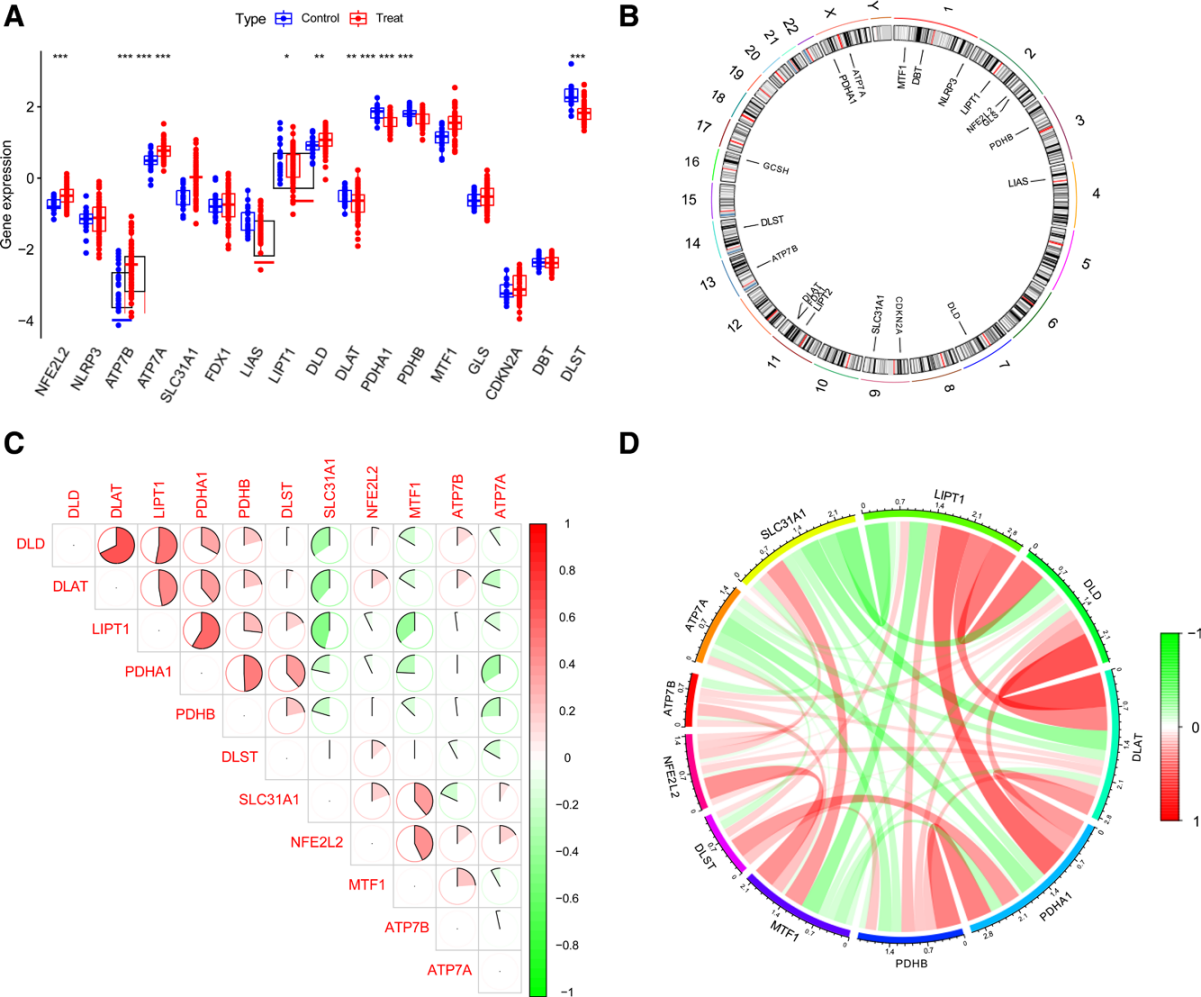
Identification of dysregulated DECAGs in CES. (A) Box plots of 11 DECAGs between samples with CES and control samples, with red representing the CES group and blue representing the control group, *P value < .05, **P value < .01, ***P-value < .001. (B) The chromosomal location loop of 19 cuproptosis-related genes. (C, D) The correlation between every 2 DECAGs, red represents the positive correlation and green represents the negative correlation, and the darker the color, the stronger the correlation. CES = cardioembolic stroke, DECAGs = differentially expressed cuproptosis-associated genes.

A total of 21,653 CES-associated genes were identified, all of which interfaced with 19 cuproptosis genes. Notably, 11 cuproptosis-associated genes (*NFE2L2, ATP7B, ATP7A, SLC31A1, CDKN2A, DLD, DLAT, PDHA1, PDHB, MTF1*, and *DLST*) were differentially expressed in CES cells (Fig. 2A). Using the “corrplot” R language, the chromosomal positions of 19 DECAGs were plotted on a ring plot (Fig. 2B). The correlation analysis of 11 DECAGs using the “circlize” R language showed that *DLAT* had the most significant positive correlation with *DLD*, and *SLC31A1* had the most significant negative correlation with *LIPT1* (Fig. 2C, D).

### 3.2. Correlation analysis of DECAGs immunoinfiltration

**Figure 3.**
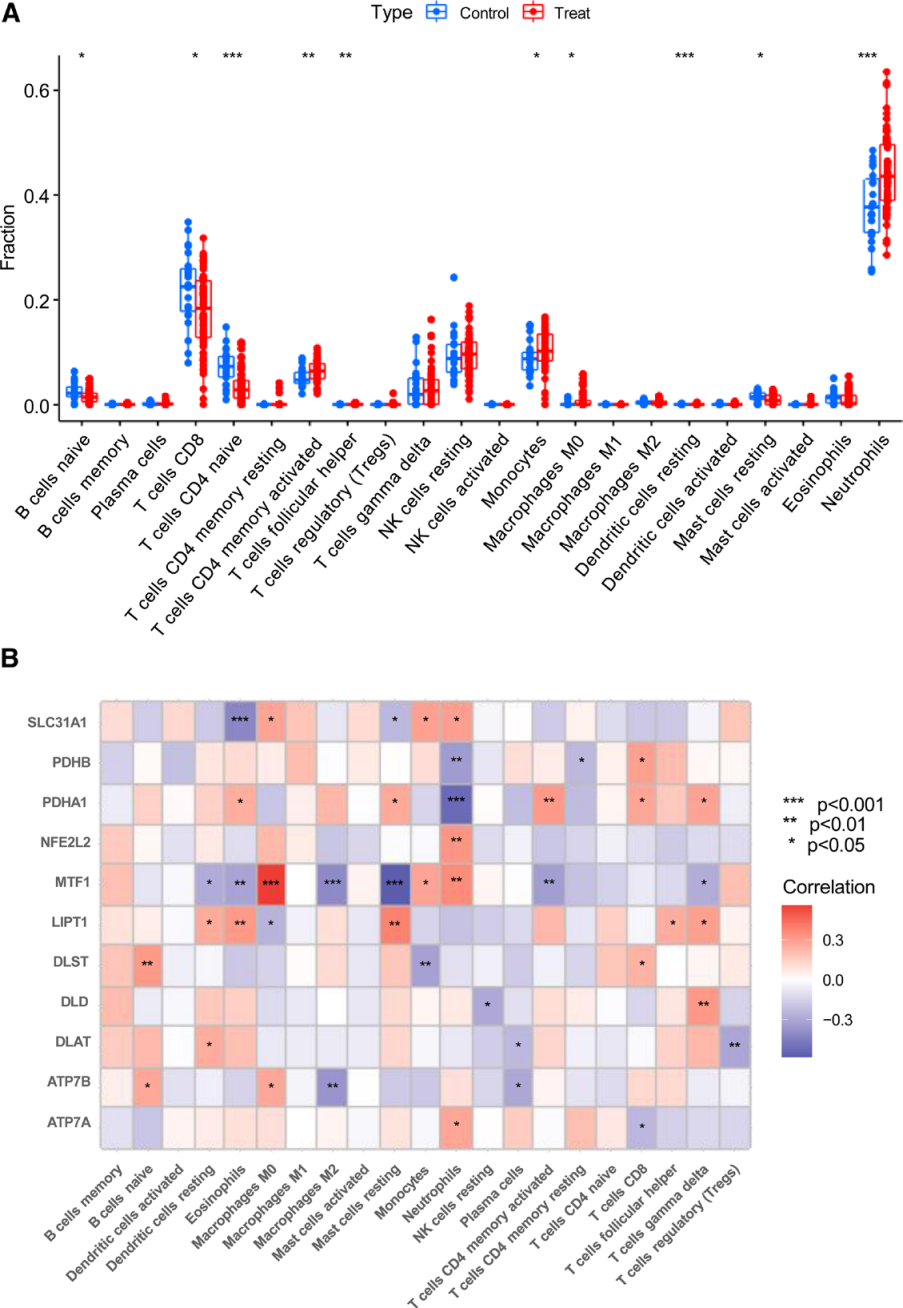
Correlation analysis of DECAGs immunoinfiltration. (A) The difference box of immunoinfiltrating cells between samples with CES and control samples, red represents the CES group, blue represents the control group, *P < .05, **P < .01, ***P < .001. (B) The heat map of the difference analysis of immune infiltrating function between samples with CES and control samples, red represents positive correlation, blue represents negative correlation, the darker the color, the more significant the correlation, and vice versa, the smaller the correlation.*P < .05, **P < .01, ***P < .001. CES = cardioembolic stroke, DECAGs = differentially expressed cuproptosis-associated genes.

Immunoinfiltration analysis showed that naive CD4^+^ T cells, resting dendritic cells, and neutrophils were significantly different between samples with CES and control samples (*P* < .001). Additionally, activated memory CD4^+^ T cells and regulatory T cells were significantly different between samples with CES and control samples (*P* < .01). Moreover, immune CD8^+^ T cells, naïve B cells, monocytes, M0 macrophages, and resting mast cells significantly differed between CES samples and control samples (*P* < .05) (Fig. 3A). Furthermore, immunoinfiltration analysis showed that *SLC31A1, PDHB, PDHA1, MTF1, LIPT1, DLST, DLD, DLAT, ATP7B*, and *ATP7A* were significantly correlated with 1 or more cell types and their functions in immunoinfiltration (Fig. 3B). These results suggest that DECAGs may be a key factor in regulating the molecular and immunoosmotic status in samples with CES.

### 3.3. Unsupervised clustering analysis of CES samples

**Figure 4.**
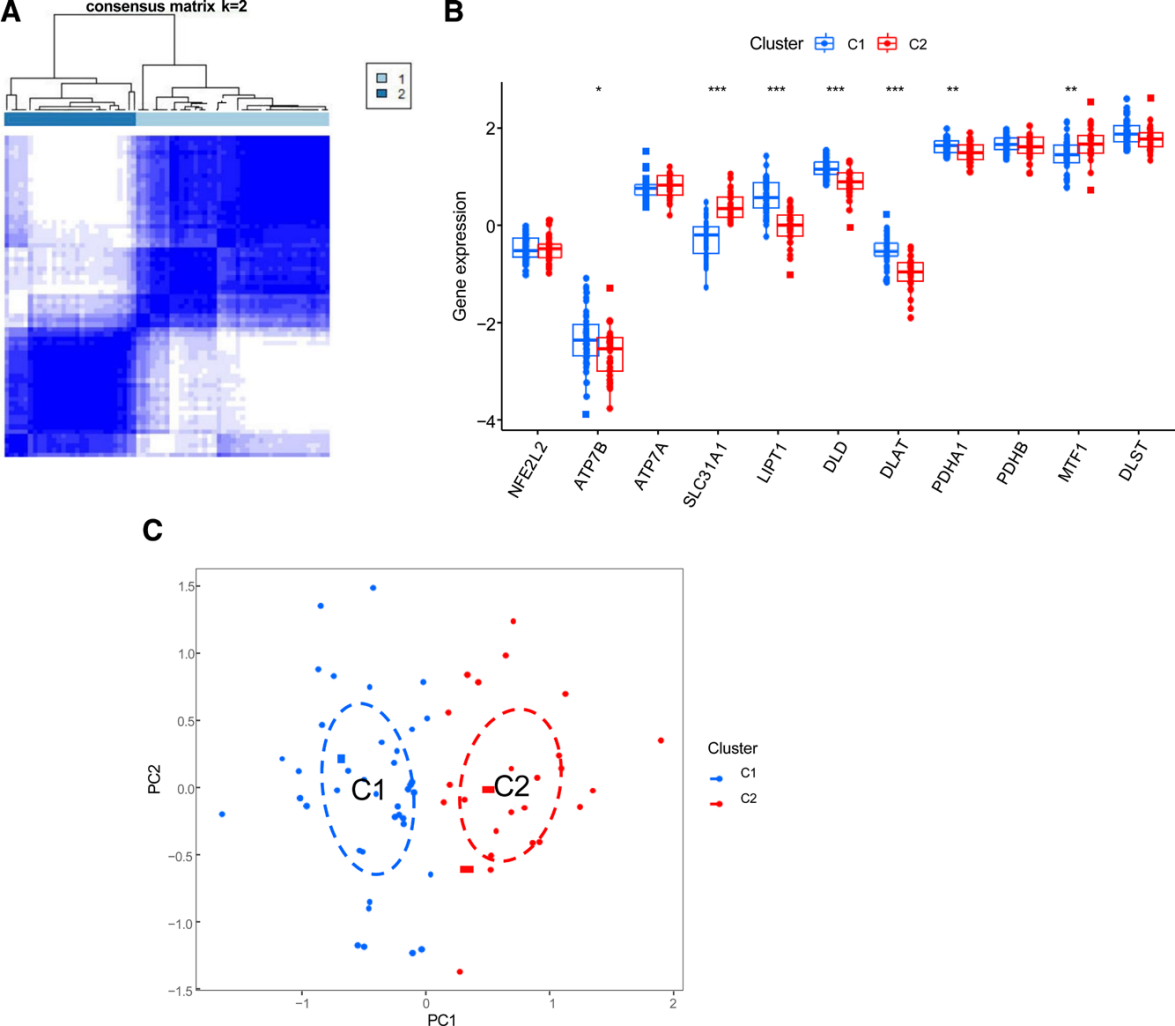
Unsupervised clustering analysis. (A) When k = 2, the matrix heat map is neatly classified. (B) The expression of 11 DECAGs between Cluster1 and Cluster2. *P < .05, **P < .01, ***P < .001. (C) The PCA diagram. DECAGs = differentially expressed cuproptosis-associated genes.

To clarify the expression patterns of CES and cuproptosis-related genes, we used a consensus clustering algorithm to group 69 CES samples based on the expression profiles of the 11 DECAGs. When k = 2, the cumulative distribution function value was the largest, and clustering was optimal. We eventually divided the 69 CES samples into 2 groups: Cluster 1 (n = 41) and Cluster 2 (n = 28) (Fig. 4A). A differential expression analysis of these 2 groups revealed that certain genes among the DECAGs exhibited varying expression levels across the 2 subtypes. Specifically, *ATP7B, LIPT1, DLD, DLAT*, and *PDHA1* were upregulated in group Cluster1, whereas SLC31A1 and MTF1 were upregulated in group Cluster 2 (Fig. 4B). Principal component analysis showed that the expression levels of DECAGs could effectively distinguish between Cluster 1 and Cluster 12 (Fig. 4C).

### 3.4. Analysis of cuproptosis-related immune infiltration and GSEA enrichment between subgroups

**Figure 5.**
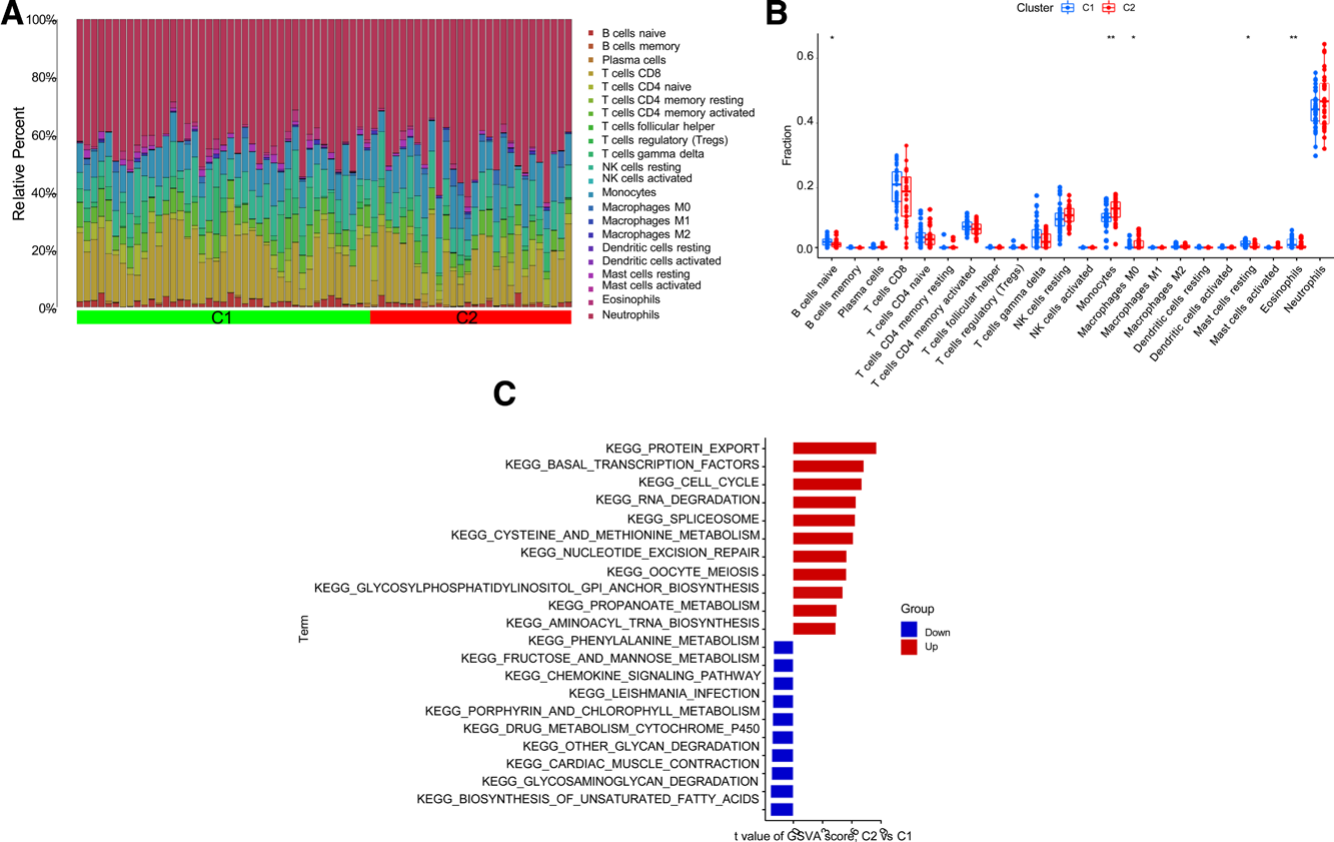
Analysis of cuproptosis-related immune infiltration and GSEA enrichment between subgroups. (A) The immunoinfiltration heat map with expression differences between Cluster1 and Cluster2. (B) The boxplot of immunoinfiltrating cells with different expression between Cluster1 and Cluster2, with blue representing group Cluster1 and red representing group Cluster2, *P < .05, **P < .01. (C) Histogram of GSEA enrichment analysis between Cluster1 and Cluster2. Red represents the path upregulated in Cluster2, blue represents the path upregulated in Cluster1, abscissa represents the score, and ordinate represents the path name.

The result files of typing and immune cell infiltration were integrated using the “Limma” R language. The results of immunoosmotic analysis showed that the immune microenvironment of Cluster 1 and Cluster 2 changed (Fig. 5A); immune cell monocytes and eosinophils showed significant differences between types Cluster 1 and Cluster 2 (*P* < .01), and naive B cells, M0 macrophages, and resting mast cells also differed between Cluster 1 and Cluster 2 (*P* < .05). Cluster 1 exhibited higher proportions of resting mast cells, naive B cells, and eosinophils, whereas Cluster 2 had higher proportions of monocytes and M0 macrophages (Fig. 5B). GSEA is a method used to determine whether a predefined set of genes shows statistically significant and consistent differences between 2 biological states.^[22]^ The GSVA algorithm was executed using the “Limma” R language according to *P* < .05. The threshold of 1 was used to determine the pathway of significant enrichment. Important signaling pathways in CES were identified based on the GSEA results. These pathways included the protein export, propionate metabolism, cell cycle, nucleotide excision repair, cysteine and methionine metabolism-reference, basal transcription factors, aminoacyl-tRNA biosynthesis-reference, chemokine signaling, cardiac muscle contraction, biosynthesis of unsaturated fatty acids, and glycosaminoglycan degradation pathways (Fig. 5C).

### 3.5. Gene module screening and co-expression network construction

**Figure 6.**
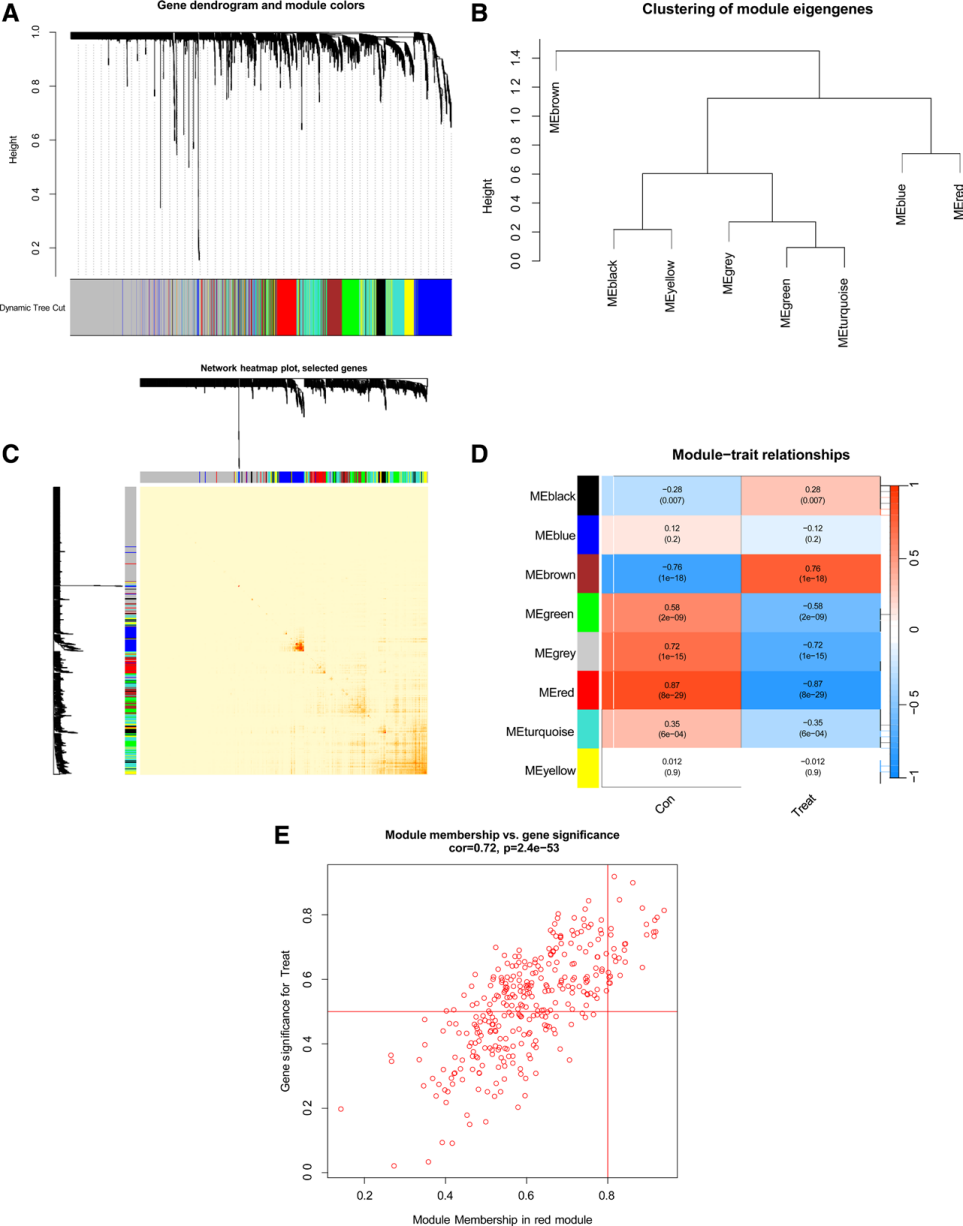
(A) Clustering tree diagram of co-expression modules. Different colors represent different co-expression modules, and the larger the module area, the more corresponding genes. (B) Correlation of module feature gene clustering. In the figure, MEblack and MEyellow are closely correlated, MEblue and MEred are closely correlated, and MEgreen and MEturquoise are closely correlated. (C) Representative heat map of the correlation between 8 modules. The darker the gene color between modules, the more significant the gene correlation. (D) Correlation analysis between module characteristic genes and clinical status. Each row represents a module, and each column represents a clinical state. It is necessary to find the module with the smallest *P* value. As the module most relevant to the disease, the red module in the figure has the smallest *P* value. (E) Scatter plot between the gene significance of module members in the MEred module and cardioembolic stroke, with MM representing the correlation between genes and modules and GS representing the importance of genes. Genes in the MEred module are filtered according to MM and GS conditions to get the core genes of the module.

To identify the core genes for CES consensus cluster typing, we used the WGCNA algorithm to establish co-expression networks and modules for normal and CES samples. We calculated the variance of each gene expression in GSE5829 and selected the top 25% of the genes with the largest variance for further analysis. The dynamic cutting algorithm was used to obtain 8 different core-presentation modules, each denoted by a unique color; the heat map of the TOM is presented in Figure 6A–C. Subsequently, these genes in the 8 color modules were successively applied to analyze the similarity and adjacency of modular-clinical feature (control and CES) co-expression. Among these modules, the MEred module, including 326 genes (Fig. 6D), had the lowest adjusted *P* value (*P* < 8e − 29), indicating that it was most closely related to CES. Additionally, we observed a positive relationship between the MEred module and module-related genes (Fig. 6E).

**Figure 7.**
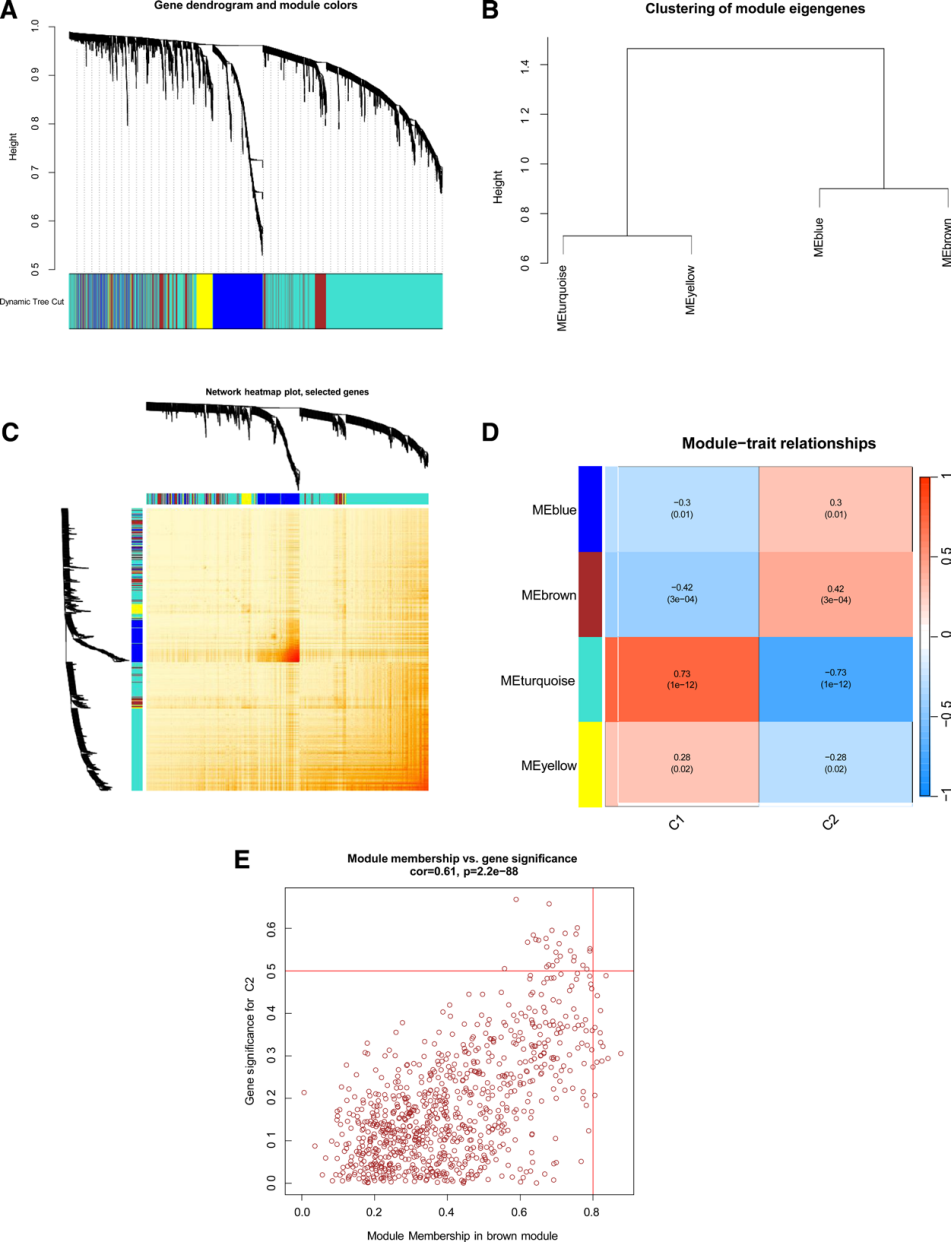
Co-expression network of differentially expressed genes between the 2 cuproptosis clusters. (A) Clustering tree diagram of the co-expression module. Different colors represent different co-expression modules, and the larger the module area, the more corresponding genes. (B) The representation of module characteristic gene clustering, in which MEturquoise and MEyellow are closely correlated, and MEblue and MEbrown are closely correlated. (C) The representative heat map of the correlation between the 4 modules, in which the darker the gene color between the modules, the more significant the gene correlation is. (D) Correlation analysis between module characteristic genes and clinical status. The abscess represents sample typing, ordinate represents module name, different colors represent different modules, number at the top of the figure represents the correlation coefficient, and number in square brackets at the bottom represents the correlation test *P* value. It is necessary to find the module with the smallest *P* value as the most relevant module for typing. In the figure, the brown module has the smallest *P* value.

In addition, we used the WGCNA algorithm to conduct typing co-expression analysis to identify typing-related modules. Four modules containing 5413 genes were identified as important; the heat map depicts the TOM of all module-related genes (Fig. 7A–C). The modular-clinical characteristics (Cluster 1 and Cluster 2) relationship analysis showed a high correlation between the MEbrown module (856 genes) and the CES module (Fig. 7D). Notably, correlation analysis revealed that the MEbrown module was significantly related to the selected module (Fig. 7E).

**Figure 8.**
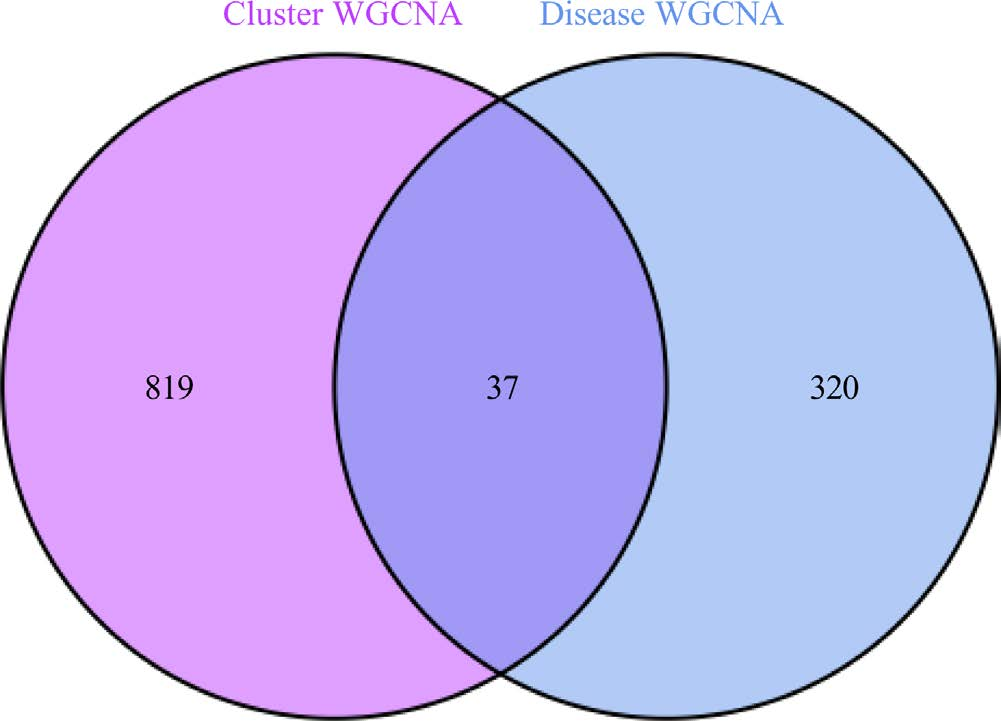
The Venn map of key genes of CES and key genes of CES typing was established, and 37 intersection genes were obtained as the core genes of CES. CES = cardioembolic stroke, WGCNA = weighted gene co-expression network analysis.

Finally, we used the “VennDiagram” R language to establish the Venn diagram of the key genes of CES and those of CES typing, and 37 intersection genes were identified as the key genes of CES for subsequent analysis (Fig. 8).

### 3.6. Construction and assessment of machine learning models

**Figure 9.**
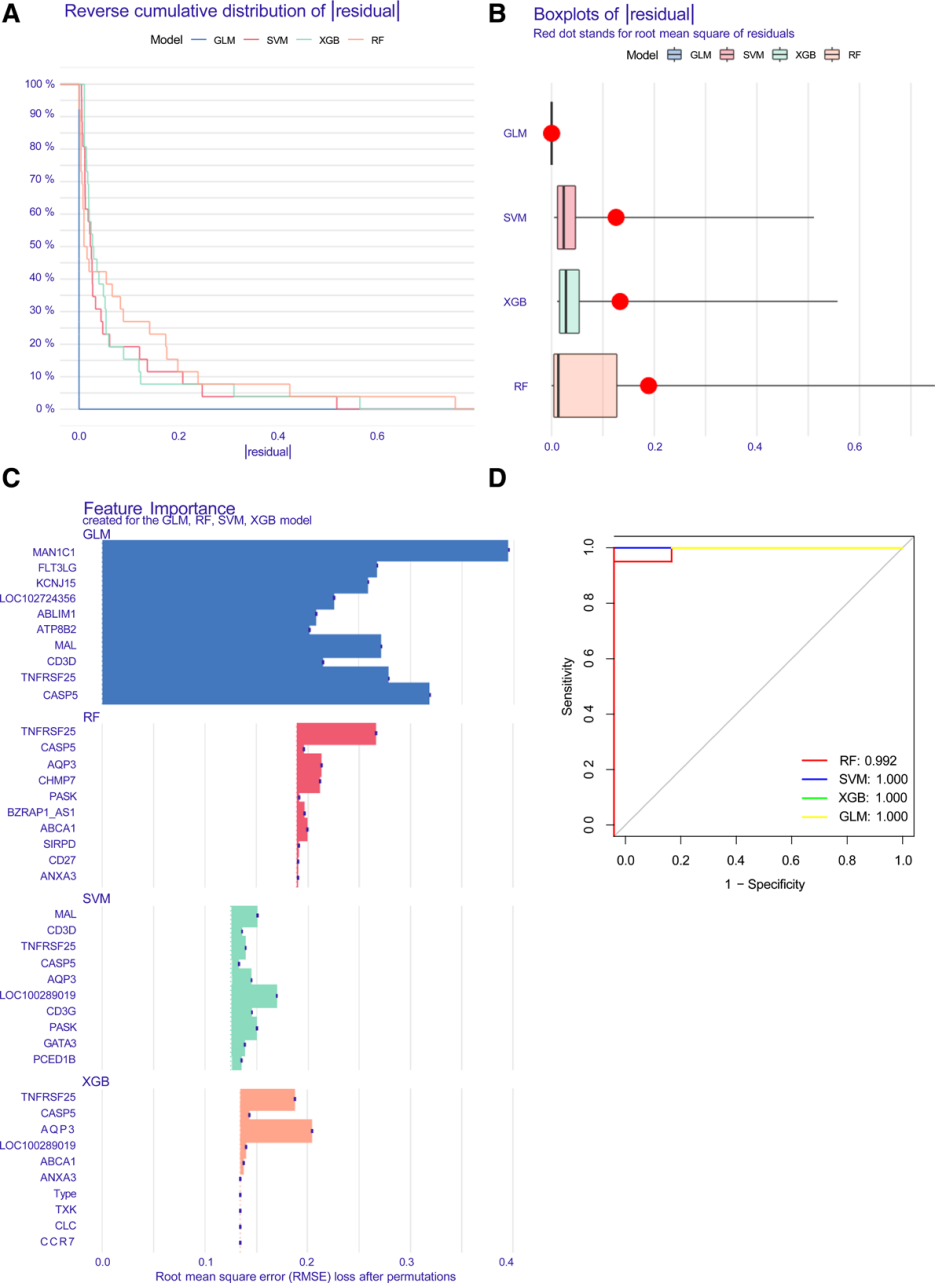
Construction and evaluation of RF, SVM, GLM, and XGB machine models. (A) Cumulative residual distribution of each machine learning model. (B) Boxplots showed the residuals of each machine learning model. Red dot represented the root mean square of residuals (RMSE). (C) The important features in RF, SVM, GLM, and XGB machine models. (D) ROC analysis of 4 machine learning models based on 5-foldcross-validation in the testing cohort. GLM = generalized linear model, ROC = receiver operating characteristic curve, RF = random forest, SVM = support vector machine, XGB = the eXtreme gradient boosting.

R software was used to construct machine learning models to analyze 37 intersecting genes. Four models: SVM, RF, XGB, and GLM, were used to identify diagnostic signature genes. The “Dalex” package was applied to interpret the 4 models and plot the residual distribution of each model in the test set. The RF and GLM machine learning models provided relatively low residuals (Fig. 9A and B). The top 10 important feature variables of each model were then ranked according to the root mean square error (Fig. 9C). In addition, the differential performances of the 4 machine learning algorithms in the test set were evaluated by calculating the ROC based on 5 cross-validations. According to the overall ROC analysis, the GLM had a higher area under the ROC curve (AUC value = 1.0) than in the RF model (Fig. 9D). These findings suggest that the GLM was more effective in differentiating between different patient clusters. Finally, the 5 most important variables (*FLT3LG, MAL, TNFRSF25, CASP5*, and *MAN1C1*) were selected from the GLM as predictive genes for further analysis.

**Figure 10.**
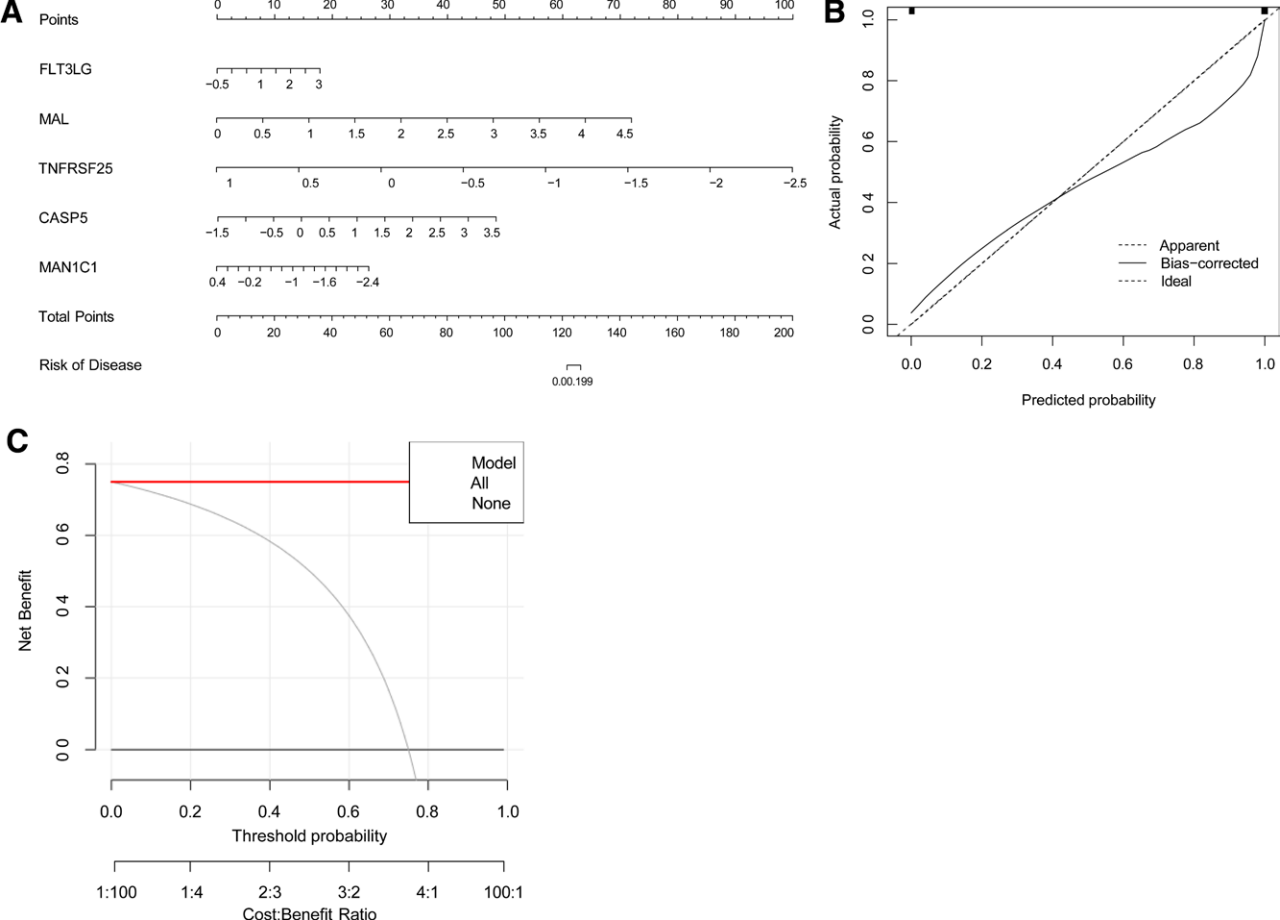
Validation of the 5-gene-based GLM model. (A) Construction of a nomogram for predicting the risk of CES clusters based on the 5-gene-based GLM model. (B, C) Construction of calibration curve (B) and DCA (C) for assessing the predictive efficiency of the nomogram model. GLM = generalized linear model.

To evaluate the predictive accuracy of the GLM model, a nomogram model was constructed (Fig. 10A and B); this nomogram demonstrated that the GLM model had high accuracy. Furthermore, we used the GLM model to construct a nomogram to assess the risk of cuproptosis clusters in 69 samples with CES (Fig. 10C). The expression levels of patient characteristic genes (*FLT3LG, MAL, TNFRSF25, CASP5*, and *MAN1C1*) were scored, and a comprehensive score was obtained to predict disease incidence.

## 4. Discussion

CES accounting for approximately 25% of ischemic strokes, is intricately linked to cardiac pathologies such as AF and valvular disease.^[23]^ Inflammation and oxidative stress are recognized drivers of CES progression.^[24]^ The inflammatory process of ischemic nerve injury is unusually complex. This process begins with reduced cerebral perfusion, followed by hypoxia, increased reactive oxygen species (ROS) in the brain (damage to vascular structure), and finally oxidative stress and activation of endothelial cells (initiating immediate immune response). Additionally, heme oxygenase-1 can degrade heme during the process, thereby releasing carbon monoxide, biliverdin IXa, and ferrous iron, which can play a role in anti-neuroinflammation and anti-apoptosis of nerve cells.^[25–30]^ Cuproptosis, distinct from apoptosis, ferroptosis, and necroptosis, is characterized by copper-dependent aggregation of lipoylated mitochondrial enzymes and proteotoxic stress. High concentrations of copper ions can induce cell death and play a role in cell survival or death under inflammatory stress conditions. This programmed cell death caused by intracellular copper overload is called copper death.^[11]^ Recent studies implicate copper overload in exacerbating ischemic injury by impairing vascular endothelial function and amplifying oxidative stress.^[11,31]^ However, the specific mechanisms behind cuproptosis and its regulatory role in CES have not been defined, and potential biomarkers of cuproptosis-associated genes in the diagnosis and treatment of CES are still lacking.

Our analysis identified 11 DECAGs, including SLC31A1, ATP7A/B, and DLAT, which regulate copper transport and mitochondrial metabolism. These genes intersect with pathways governing apoptosis (e.g., CASP5) and redox homeostasis (e.g., NFE2L2), suggesting synergistic crosstalk between cuproptosis and other cell death modalities. For instance, ATP7A knockdown exacerbates ferroptosis via SLC7A11 degradation,^[32]^ while NFE2L2 modulates antioxidant responses that may counteract copper-induced ROS accumulation.^[33]^ We then calculated the correlation between various DECAGs, and the DECAGs correlation analysis showed that some cuproptosis-associated genes exhibited obvious synergistic or antagonistic effects. The DECAGs immunoinfiltration analysis showed that *SLC31A1*, *PDHB*, *PDHA1*, *MTF1*, *LIPT1*, *DLST*, *DLD*, *DLAT*, *ATP7B*, and *ATP7A* genes were significantly correlated with 1 or more cells and their functions in immunoinfiltration. In related research reports, *SLC31A1* also increases intracellular ROS levels by inhibiting hypoxia induction factor 1 expression and the *NrF2*-dependent ROS clearance system, enhances mitochondrial oxidative phosphorylation, and induces ROS accumulation.^[34]^ In response to severe oxidative stress, *NFE2L2* induces the expression of *Klf9*, which inhibits ROS suppressor enzymes and promotes cell death.^[35]^ However, research on the mechanisms underlying the association between *SLC31A1* and CES is lacking. The adipose reduction associated with *DLAT* and *DLST* affects the decrease in mitochondrial oxidative respiration, resulting in excessive copper in the body and the upregulation of *ATF3/SPI1/SLC31A1* signal transduction, thus interfering with copper homeostasis.^[36]^ *PDHA1* and *PDHB* are located in brain mitochondria, and *PDHB* is upregulated by hypoxia induction factor 1 expression under hypoxic conditions.^[37]^ Moreover, *PDHB* affects the expression of genes related to arachidonic acid metabolism and Ras signaling pathways, such as Pla2g4a and Rsa-14-44, thereby promoting axon regeneration and acting as a positive regulator of energy generation and gene expression.^[38]^ *PDHA1* regulates cell growth and proliferation and is closely associated with cell aging.^[39]^ Downregulation of phosphorylates the catalytic alpha subunit of PDHc (PDHA) phosphorylation helps to regulate the brain acetylation system and reduction and oxidation reactions metabolism.^[40]^ Furthermore, *CDKN2A* is associated with immune complex responses and the overproduction of inflammatory cells in CES.^[41]^ *DLST* can mediate oxidative phosphorylation of myeloid cells and the expression of immunosuppressive markers, thereby regulating the immune microenvironment and preventing related inflammatory diseases.^[42]^ The *PDHA* activity is inversely proportional to the mitochondrial ATP:ADP ratio.^[43]^ The reduction in ATP7A results in abnormal copper metabolism in the mitochondria, retention of ROS, and accelerated degradation of SLC7A11, thus enhancing the oxidative stress of CRC cells and leading to iron ptosis.^[44]^ Hepatocytes reduce ATP7B activation of autophagy to prevent copper-induced apoptosis.^[45]^ Increased *ATP7A* and *ATP7B* mRNA levels lead to decreased copper content.^[46]^ *DLD* is a key gene involved in “copper dystrophy,” mainly related to the tricarboxylic acid cycle, aerobic respiration, and mitochondria-related cellular components. Abnormal *DLD* expression positively correlates with neutrophils, however, its role in immunity remains unclear.^[47]^ A small amount of Cu + enhances MTF1 expression and promotes myogenesis.^[48]^ MTF1 is involved in REDOX homeostasis and heavy metal detoxification. It works by regulating the expression of metal ion response genes.^[49]^ In this way, it protects cells needed for embryonic development in vertebrates from oxidative and hypoxic stress.

Cluster-specific DECAGs showed that *ATP7B*, *LIPT1*, *DLD*, *DLAT*, and *PDHA1* were upregulated in group Cluster1, whereas *SLC31A1* and *MTF1* were upregulated in group Cluster2 and Cluster1 showed a relatively high level of immune infiltration. Important signaling pathways in CES were identified according to the GSEA enrichment analysis results, including the protein export, cysteine and methionine metabolism-reference, chemokine signaling, cardiac muscle contraction, glycosaminoglycan degradation, and biosynthesis of unsaturated fatty acids pathways. The enriched pathways are related to inflammatory immune response, apoptosis, myocardial contraction, lipid metabolism, protein phosphorylation, oxidative damage repair, cysteine-pyruvate metabolism, and other biological processes. The Tat pathway is reportedly 1 of the protein export pathways. TAT-GDNF protects brain neurons and retinal ganglion cells from death, increases tissue Bcl XL levels, and weakens the activity of caspase-3 in the executor.^[50,51]^ The cysteine and methionine metabolism-reference pathway downregulates interleukin-6 (IL-6), IL-1B, gunshot residue, and glutathione synthetase, suggesting that it can reduce cellular inflammation and enhance antioxidant status.^[52]^ Chemokines play a chemotactic role in immune cells, regulating cell proliferation and other functions.^[53]^ Neuronal transmembrane chemokine (CX3CL1) signals through its unique receptor CX3CR1 and is expressed in microglia, thus acting as a regulator of microglia activation during brain injury or inflammatory responses.^[54]^ Interleukin-8 is a pro-inflammatory CXC chemokine that activates multiple intracellular signaling pathways downstream of CXCR1 and CXCR2 (2 cell surface G protein coupled receptors), promoting neutrophil chemotaxis and degranulation.^[55]^ Overactivity of transient receptor potential vanilloid 4 is associated with hypersystole, cardiac damage, ventricular arrhythmia, and AF. Overactivation of transient receptor potential vanilloid 4 leads to an increase in calcium, which predisposes cardiomyocytes to arrhythmia.^[56]^ Glycosaminoglycans regulate physiological processes by binding to various immunoactive proteins and enhance the formation of ROS in interleukin-8-induced neutrophils.^[57]^ Omega-3 polyunsaturated fatty acids have the strongest immunomodulatory activity and have anti-inflammatory properties, contributing to the treatment of inflammatory and autoimmune diseases.^[58]^ In summary, it can be concluded that the enriched pathways are more relevant than the inflammatory immune response.

In this study, the molecular patterns, regulatory mechanisms, and enrichment pathways associated with CES DECAG were comprehensively analyzed. With a combination of immunoinfiltration analysis of CES and correlation results of cuproptosis gene immunoinfiltration, we observed that CES showed higher levels of CD4 naive T cells, neutrophils, memory activated CD4 T cells, and regulatory T cells. The infiltration levels of CD8 cells, monocytes, M0 macrophages, and naive B cells can be concluded from the above. The highly expressed genes and enriched pathway of cuproptosis may be the key factors regulating molecular and immune response in patients with CES, most of which are associated with inflammation. This is consistent with the research by Krishnan et al.^[59–61]^

In this study, we developed RF, SVM, XGB, and GLM models. From the overall ROC analysis, the GLM model had a higher AUC value than the other models (AUC = 1.0), suggesting that GLM-based machine learning has satisfactory performance in predicting CES subtypes.

By combining WGCNA and machine learning (ML), we identified 5 diagnostic genes (FLT3LG, MAL, TNFRSF25, CASP5, and MAN1C1) with high predictive accuracy (AUC = 1.0). WGCNA prioritized the MEbrown module, enriched in inflammation-related genes (e.g., TNFRSF25, CASP5), which aligns with prior work linking TNF receptor superfamily members to endothelial dysfunction. At present, some studies on 5 characteristic genes have been published. For example, Cueto et al^[62]^ demonstrated specific inflammation in CDC1 induced by FLT3LG. Bernard et al^[63,64]^ demonstrated that MAL may enhance toll-like receptor 2 (TLR2) signaling and induce IL-6 or NF jB in TLR2 signaling. Low dose TLR2 mediated by MAL in the early stages of the disease may initiate a protective immune response, preventing bacterial growth.

*TNFRSF25* is an important mediator of several chronic immune diseases and actively participates in the mucosal homeostasis of the immune pathway. It plays a protective role by reducing the severity of acute inflammatory responses and promoting tissue repair.^[65]^ *TNFRSF25* activates inflammatory immune-related pathways (such as NF-κB and phosphoinositide 3-kinase-AKT axis) that depend on the synergistic interaction of T cell receptors and IL-2 receptors.^[66]^ *CASP5* is correlated with immune cells and molecules in the LGG microenvironment.^[67]^ CASP5 enables the innate immune system to recognize a wide range of bacterial LOS/lipid A, thus potentially enhancing innate immune detection capabilities.^[68]^ IL-6 levels are associated with the transcription of inflammatory endothelial cells and the whole blood inflammasome-associated transcript *CASP5*.^[69]^ MAN1C1 is a target downregulated by TNF-α, and the activated receptor PPARϒ can mediate its anti-inflammatory effects.^[70]^ It can be inferred from the above that the use of the nomogram generated by the expression of these 5 characteristic genes may help diagnose and prevent CES through serum detection at a relatively early clinical stage, thus reducing the incidence rate and complications of CES, which is also the next research direction of this research group.

While this study provides novel insights, limitations exist. First, the small cohort size (n = 69 CES) may limit generalizability. Second, experimental validation of DECAGs in preclinical models (e.g., copper-deficient AF mice) is needed to establish causality. Third, the role of non-coding RNAs in regulating CAGs remains unexplored. Although our model’s performance requires validation in larger cohorts, the integration of multi-omics data and ML represents a robust framework for biomarker discovery in heterogeneous diseases like CES. Recent studies similarly employed WGCNA and ML to dissect immune-metabolic networks in atherosclerosis^[71]^ and AF,^[72]^ reinforcing the methodological rigor of our approach.

## 5. Conclusions

This study found through immune infiltration analysis that the molecular patterns and diagnostic biomarkers related to cuproptosis in cardiogenic stroke provide a theoretical basis for the study of cuproptosis-mediated cardiogenic stroke. However, elucidating the mechanism of cuproptosis in cardiogenic stroke remains a key focus of future research. The potential regulatory mechanism of copper death on cardiogenic stroke needs further investigation, and the strong correlation between immune inflammatory response and cardiogenic stroke deserves further confirmation in clinical biology studies. Meanwhile, more detailed clinical data is needed to confirm the performance of the predictive model. This study speculates that the abnormal expression of characteristic genes in the disease group may be related to copper ion overload or copper death.

## Acknowledgments

We would like to thank Editage (www.editage.cn) for English language editing.

## Author contributions

**Conceptualization:** Dandan Zhang, Ning Zhang.

**Data curation:** Ning Zhang.

**Formal analysis:** Qian Zhang, Yan Wang.

**Methodology:** Qian Zhang.

**Software:** Dandan Zhang.

**Writing – original draft:** Qian Zhang, Ning Zhang.

**Writing – review & editing:** Dandan Zhang, Yan Wang.
